# Synthesis of a magnetic polystyrene-supported Cu(II)-containing heterocyclic complex as a magnetically separable and reusable catalyst for the preparation of *N*-sulfonyl-*N*-aryl tetrazoles

**DOI:** 10.1038/s41598-023-30198-7

**Published:** 2023-02-24

**Authors:** Mahmoud Nasrollahzadeh, Narjes Motahharifar, Khatereh Pakzad, Zahra Khorsandi, Talat Baran, Jinghan Wang, Benjamin Kruppke, Hossein Ali Khonakdar

**Affiliations:** 1 Department of Chemistry, Faculty of Science, University of Qom, PO Box 37185-359, Qom, Iran; 2 Max Bergmann Center of Biomaterials, Institute of Materials Science, Technische Universität Dresden, 01069 Dresden, Germany; 3 Department of Chemistry, Faculty of Science and Letters, Aksaray University, 68100 Aksaray, Turkey; 4 Department of Materials Science and Engineering, Research Institute of Advanced Materials, Seoul National University, Seoul, 08826 Republic of Korea; 5 Department of Processing, Iran Polymer and Petrochemical Institute, Tehran, Iran

**Keywords:** Catalysis, Materials chemistry, Organic chemistry, Chemical synthesis

## Abstract

In this work, a cost-effective, environmentally friendly, and convenient method for synthesizing a novel heterogeneous catalyst via modification of polystyrene using tetrazole-copper magnetic complex [Ps@Tet-Cu(II)@Fe_3_O_4_] has been successfully developed. The synthesized complex was analyzed using TEM (transmission electron microscopy), HRTEM (high resolution-transmission electron microscopy), STEM (scanning transmission electron microscopy), FFT (Fast Fourier transform), XRD (X-ray diffraction), FT-IR (Fourier transform-infrared spectroscopy), TG/DTG (Thermogravimetry and differential thermogravimetry), ICP-OES (Inductively coupled plasma-optical emission spectrometry), Vibrating sample magnetometer (VSM), EDS (energy dispersive X-ray spectroscopy), and elemental mapping. *N*-Sulfonyl-*N*-aryl tetrazoles were synthesized in high yields from *N*-sulfonyl-*N*-aryl cyanamides and sodium azide using Ps@Tet-Cu(II)@Fe_3_O_4_ nanocatalyst. The Ps@Tet-Cu(II)@Fe_3_O_4_ complex can be recycled and reused easily multiple times using an external magnet without significant loss of catalytic activity.

## Introduction

Catalysts have been used widely for chemical transformations; especially organic reactions. However, the effective separation of homogeneous catalysts is a remarkable scientific and engineering challenge. The use of heterogeneous catalysts is an efficient method to solve this problem. Heterogeneous catalysts have many advantages such as easy recovery and recyclability from the reaction media using centrifugation, filtration, and magnetic alteration^1–10^. Heterogeneous catalysts can be immobilized on various supports such as graphene, polymers, magnetic nanoparticles, zeolite, carbon, mesoporous silica, and silica sol–gels^11–26^. In recent decades, polymer-based supports have been studied extensively due to their several specifications, well-controlled structure, and ease of functionalization^15–21^. For example, polystyrene (PS) is one of the extensively used polymers. The introduction of various functions to PS produces effective nanocomposite supports for heterogeneous catalysts^17,20,21^.

Nanomaterials are one of the most important types of compounds, which can be applied in different fields^27–37^. Metal nanoparticles (MNPs) are the most important nanomaterials^38–45^. MNPs have most of the particular features of an appropriate catalyst, including low price, great activity, high surface area, low toxicity, significant thermal stability, simple recoverability, and excellent recyclability^46–56^. From this perspective, MNPs-supported catalysts are associated with green chemistry and sustainability^57–65^. Among various MNPs, copper-based catalysts represent considerable catalytic activities. Copper has received wide attention as an effective transition metal owing to its remarkable advantages such as numerous sources, low cost, diversity, low environmental hazards, and extensive applications^66–72^. In recent years, scientists have tried to decrease the costs of organic reactions by replacing palladium with cheap metals such as copper^73–75^.

Today, researchers are paying a lot of attention to the field of catalysis^76–82^. Recently, magnetic NPs have been widely used as catalyst supports for different organic transformations^13,18,20,57^. The most important features of magnetic nanocatalysts include their high surface-to-volume ratio, which leads to high catalytic activities, high dispersion, and excellent stability. Moreover, these catalysts contain the green advantage of suitable and efficient recyclability, owing to their simplicity of separation using a magnet. Catalysts supported on super magnetic NPs have successfully catalyzed various organic reactions^58,59^. Among heterogeneous catalysts, magnetite/polymer nanocomposite is one of the most effective nanocomposites. Fe_3_O_4_ NPs dispersed on polymer surfaces are superparamagnetic catalysts in various chemical reactions^17^.

Tetrazole is an important synthetic compound with wide applications in various fields such as pharmacology, biochemistry, medicinal chemistry, photography, and imaging chemicals. In fact, various tetrazoles; especially 5-substituted 1*H*-tetrazoles and aminotetrazoles have been applied to synthesize biologically active compounds in recent years^13,83–85^. The [2 + 3] cycloaddition reaction is a conventional method for the synthesis of tetrazoles. Given the medicinal applications of tetrazoles, different synthetic methodologies have been widely developed for their synthesis.^13,66,67,85^.

Among tetrazoles, aminotetrazoles have received much attention because of their wide-ranging applications. However, the lack of convenient methods for the synthesis of these compounds or their derivatives such as *N*-sulfonyl-*N*-aryl tetrazoles strongly restricts their potential medical applications^66,67,85^. Thus, it is desirable to develop a convenient and efficient method for the synthesis of *N*-sulfonyl-*N*-aryl tetrazoles.

**Scheme 1:**
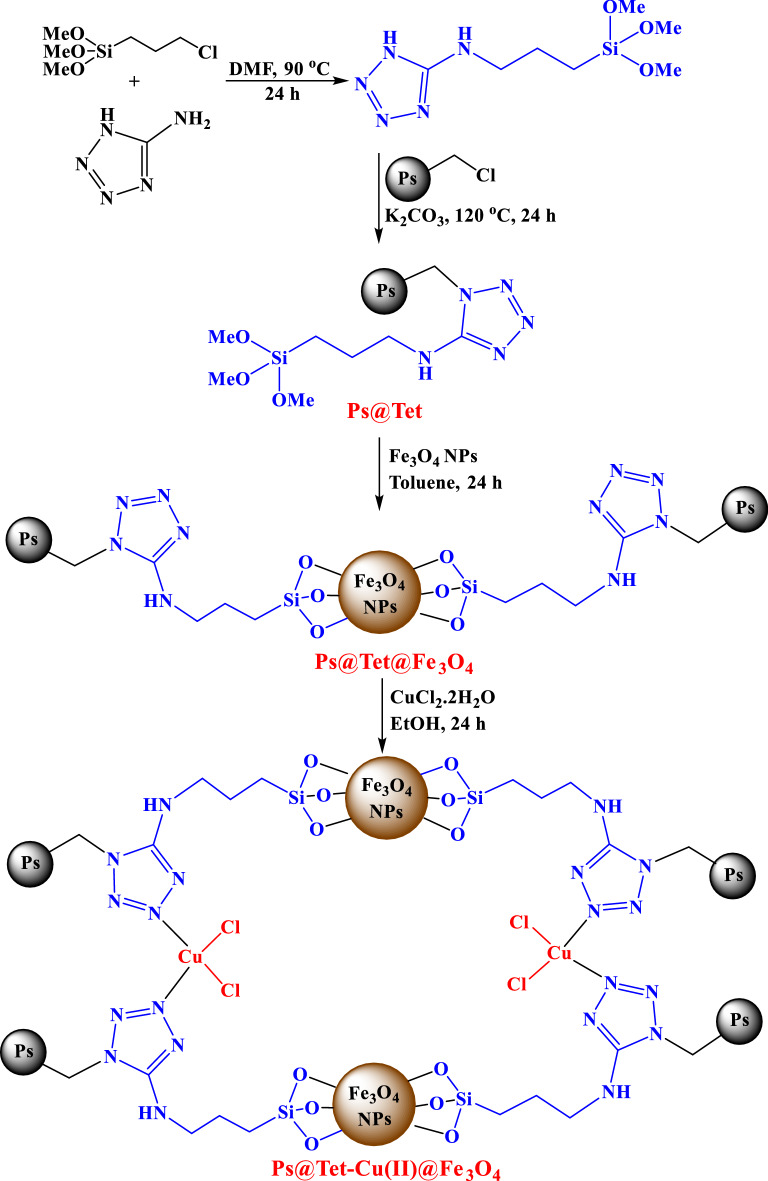
Synthesis of Ps@Tet-Cu(II)@Fe_3_O_4_.

**Scheme 2:**
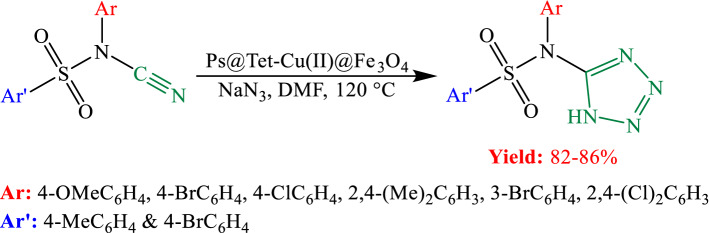
Synthesis of *N*-sulfonyl-*N*-aryl tetrazoles.

Following our research on the progress of modern catalytic systems, in this study, copper NPs immobilized on magnetic tetrazole‐functionalized polystyrene [Ps@Tet-Cu(II)@Fe_3_O_4_] have been investigated as a highly effective catalyst (Scheme 1). After the characterization of the synthesized complex by various techniques, the catalytic activity of the complex in the synthesis of *N*-sulfonyl-*N*-aryl tetrazoles was studied (Scheme 2).

## Experimental

### Instruments and reagents

TEM, STEM, and NMR spectra were recorded on JEM-F200 JEOL, JEM-F200-TFEG-JEOL Ltd, and Bruker Avance DRX 600 MHz instruments, respectively. The FT-IR spectra and XRD patterns of the samples were obtained using a Perkin Elmer 100 spectrophotometer and a Philips model PW 1373 diffractometer, respectively. The elemental compositions of the synthesized nanoparticle were determined by EDS coupled with Map. STA 1500 Rheometric-Scientific conducted TGA measurements under N_2_ flow. VSM analysis was performed using a magnetometer at 298 K (LBKFB).

### Synthesis of Ps@Tet-Cu(II)@Fe_3_O_4_

In a 250 mL beaker, a solution of 5-amino-1*H*-tetrazole (5 mmol), TMOS [(3-chloropropyl)trimethoxysilane)] (5 mmol) in DMF (60 mL) solvent was stirred for 24 h at 90 ℃. Chloromethylated polystyrene (2 g) and potassium carbonate (5 mmol) were then added to the reaction media, which was stirred for another 24 h at 120 ℃. After cooling the reaction mixture, the obtained Ps@Tet was filtrated, washed with DMF, and dried at 70 °C. Afterward, 1 g of Ps@Tet, 1.5 g of Fe_3_O_4_ NPs, and 50 mL of toluene were mixed vigorously under reflux conditions for 24 h. The synthesized Ps@Tet@Fe_3_O_4_ was then separated using an external magnet, washed with toluene, and dried at 70 °C. In the next step, 1 g of the obtained Ps@Tet@Fe_3_O_4_ and 0.5 g of CuCl_2_.6H_2_O were mixed constantly in 50 mL of ethanol solvent at 85 °C for one day. Upon completion of the reaction, the synthesized magnetic complex Ps@Tet-Cu(II)@Fe_3_O_4_ was separated using a magnet, washed with EtOH, and dried at 70 °C (Scheme 1).

### General process for the synthesis of *N*-sulfonyl-*N*-aryl tetrazoles

In a 50 mL beaker, *N*-sulfonyl-*N*-aryl cyanamide (1 mmol), NaN_3_ (1.5 mmol), and Ps@Tet-Cu(II)@Fe_3_O_4_ (0.05 g) catalyst were continuously mixed in DMF (10 mL) solvent at 120 ℃. The progress of the reaction was followed by TLC. After completion of the reaction, the magnetic catalyst was separated by an external magnet. Afterward, 25 mL of hydrochloric acid (2 N) and 25 mL of ethyl acetate were added to the reaction mixture, which was then stirred vigorously. After the separation of the organic phase, the aqueous phase was extracted by ethyl acetate (25 mL) three times and the organic layer was concentrated. The product was then purified by recrystallization from ethanol. All products were identified by NMR and FT-IR spectroscopy^66,67,85^.

### Characterization data of new product

#### 4-Bromo-*N*-(3-bromophenyl)-*N*-(1*H*-tetrazol-5-yl)benzenesulfonamide

FT-IR (KBr, cm^−1^) 3445, 3137, 1632, 1576, 1468, 1398, 1364, 1232, 1171, 966, 813, 818, 747, 690, 608, 577, 548, 502; ^1^H NMR (600 MHz, DMSO-*d*_*6*_) *δ*_H_ = 7.83 (d, *J* = 8.6 Hz, 2H), 7.73 (d, *J* = 8.6 Hz, 2H), 7.50 (d, *J* = 8.0 Hz, 1H), 7.36 (s, 1H), 7.30 (t, *J* = 8.0 Hz, 1H), 7.22 (d, *J* = 8.0 Hz, 1H); ^13^C NMR (150 MHz, DMSO-*d*_*6*_) *δ*_C_ = 159.2, 140.5, 137.1, 132.1, 131.0, 130.6, 130.1, 129.8, 127.7, 126.2, 121.2; Anal. Calcd for C_13_H_9_Br_2_N_5_O_2_S: C, 34.01; H, 1.98; N, 15.25. Found: C, 34.13; H, 2.12; N, 15.37.

## Result and discussion

### Characterization of Ps@Tet-Cu(II)@Fe_3_O_4_

**Figure 1:**
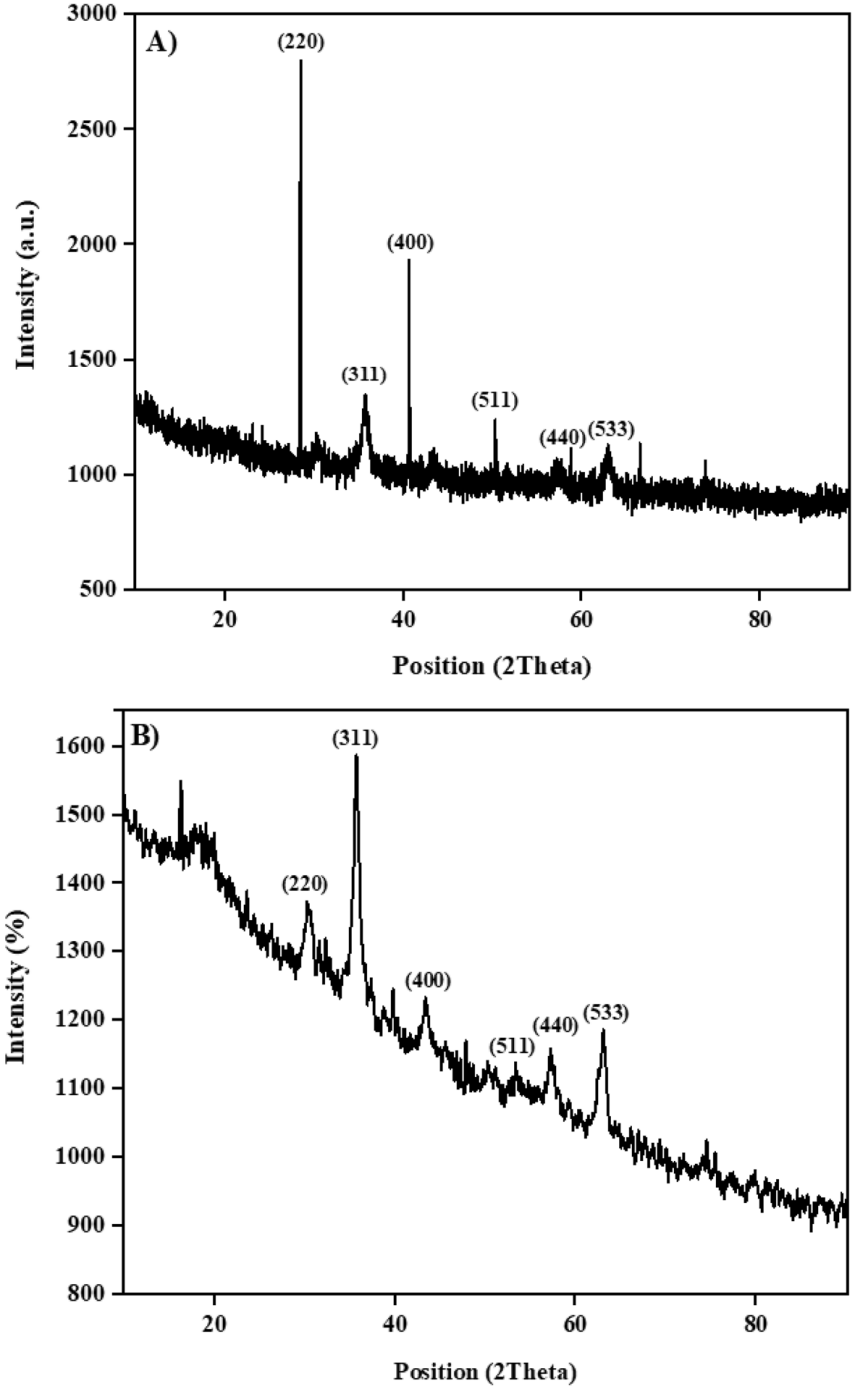
XRD powder pattern of Ps@Tet@Fe_3_O_4_ (**A**) and Ps@Tet-Cu(II)@Fe_3_O_4_ (**B**).

The XRD patterns of the synthesized Ps@Tet@Fe_3_O_4_ and Ps@Tet-Cu(II)@Fe_3_O_4_ complex are illustrated in Fig. 1. The XRD patterns demonstrate the presence of Fe_3_O_4_ NPs with diffraction angles of 30.2°, 35.8°, 43.5°, 53.7°, 57.2°, and 62.8°, which are assigned to the crystal planes of (220), (311), (400), (511), (440), and (533), respectively^67^.

**Figure 2:**
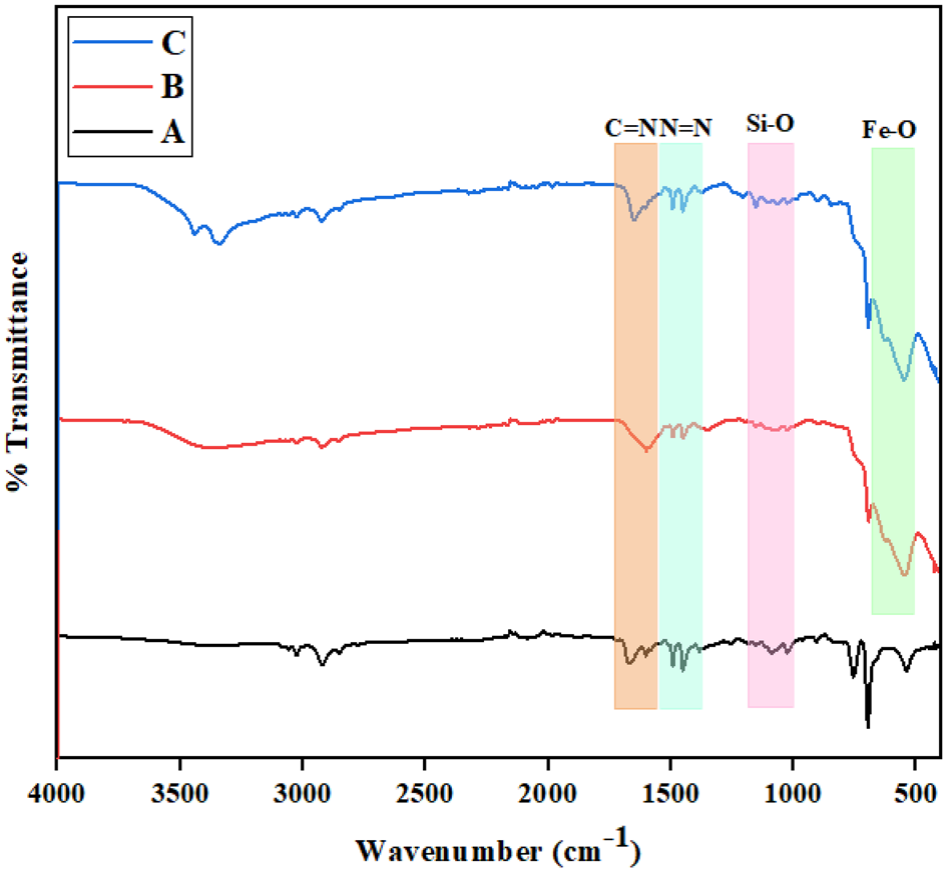
FT‐IR spectra of Ps@Tet (**A**), Ps@Tet@Fe_3_O_4_ (**B**) and Ps@Tet-Cu(II)@Fe_3_O_4_ (**C**).

FT-IR analysis was applied to confirm the presence of functional groups in complex interactions. The FT-IR spectra of the synthesized Ps@Tet, Ps@Tet@Fe_3_O_4_ and Ps@Tet-Cu(II)@Fe_3_O_4_ complex are illustrated in Fig. 2. The peaks at around 1153 cm^-1^, 1492 cm^-1^, 1650 cm^-1^, and 2922 cm^-1^ correspond to Si–O, N=N, C=N, and C–H (sp^3^) stretching vibrations, respectively. In addition, the peaks at 550 cm^−1^ and 3300–3450 cm^-1^ are due to the Fe–O bond stretching and O–H functional groups of Fe_3_O_4,_ respectively^67^.

**Figure 3:**
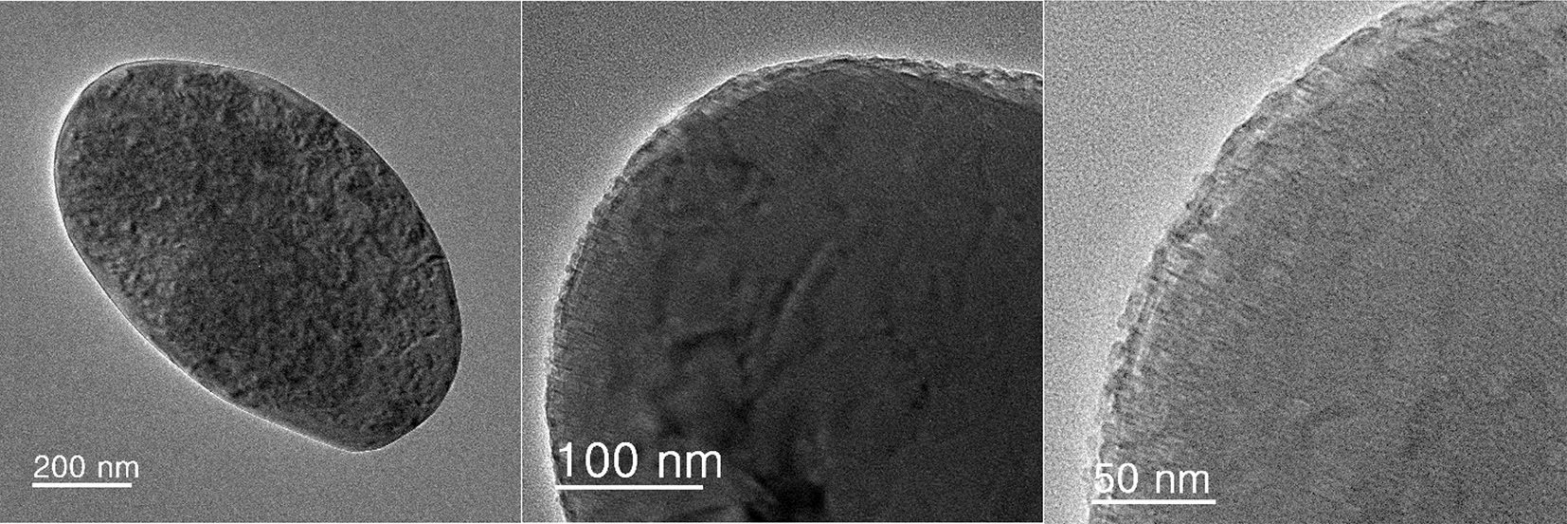
TEM images of Ps@Tet.

**Figure 4:**
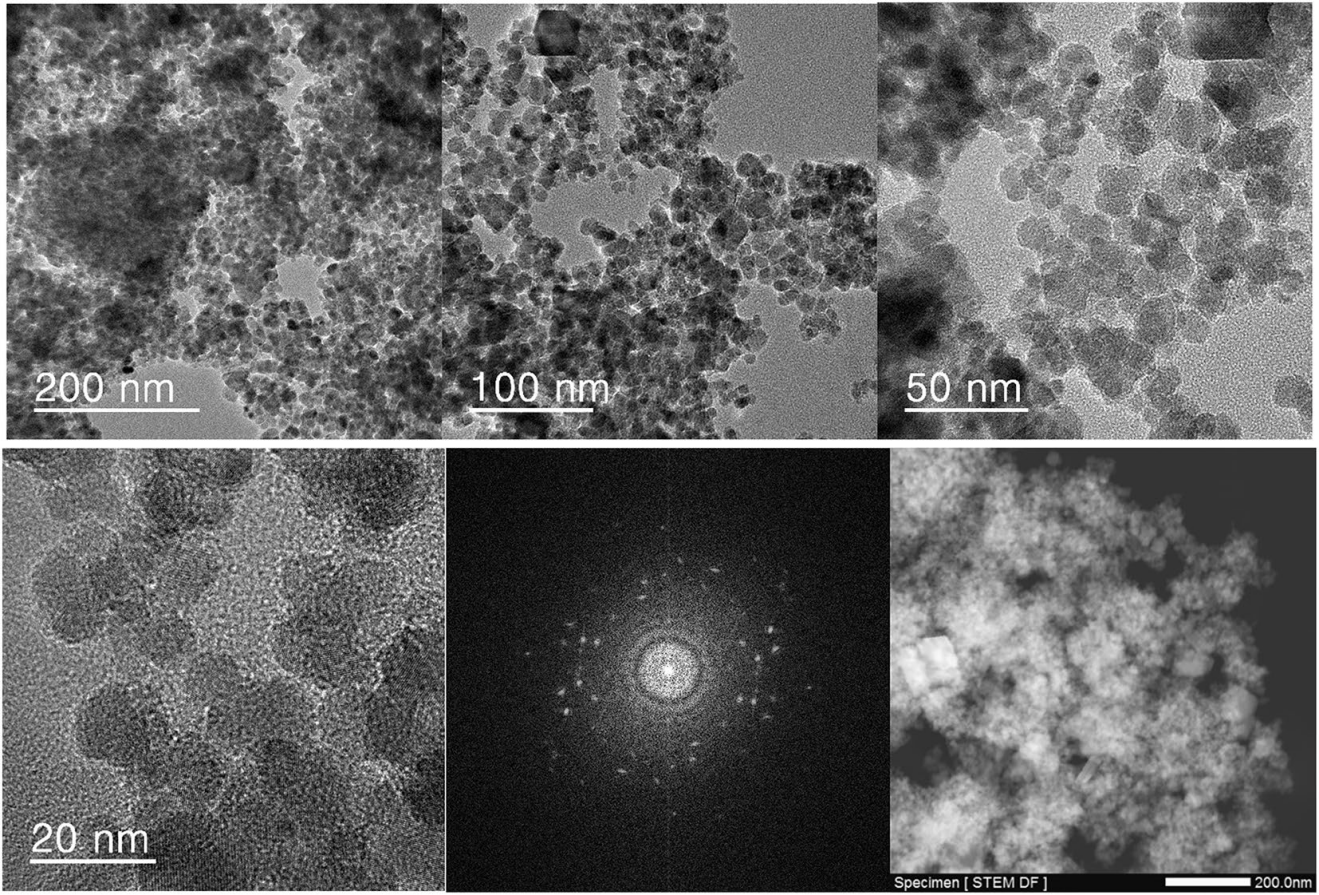
TEM, HRTEM, FFT and STEM images of Ps@Tet@Fe_3_O_4_.

**Figure 5:**
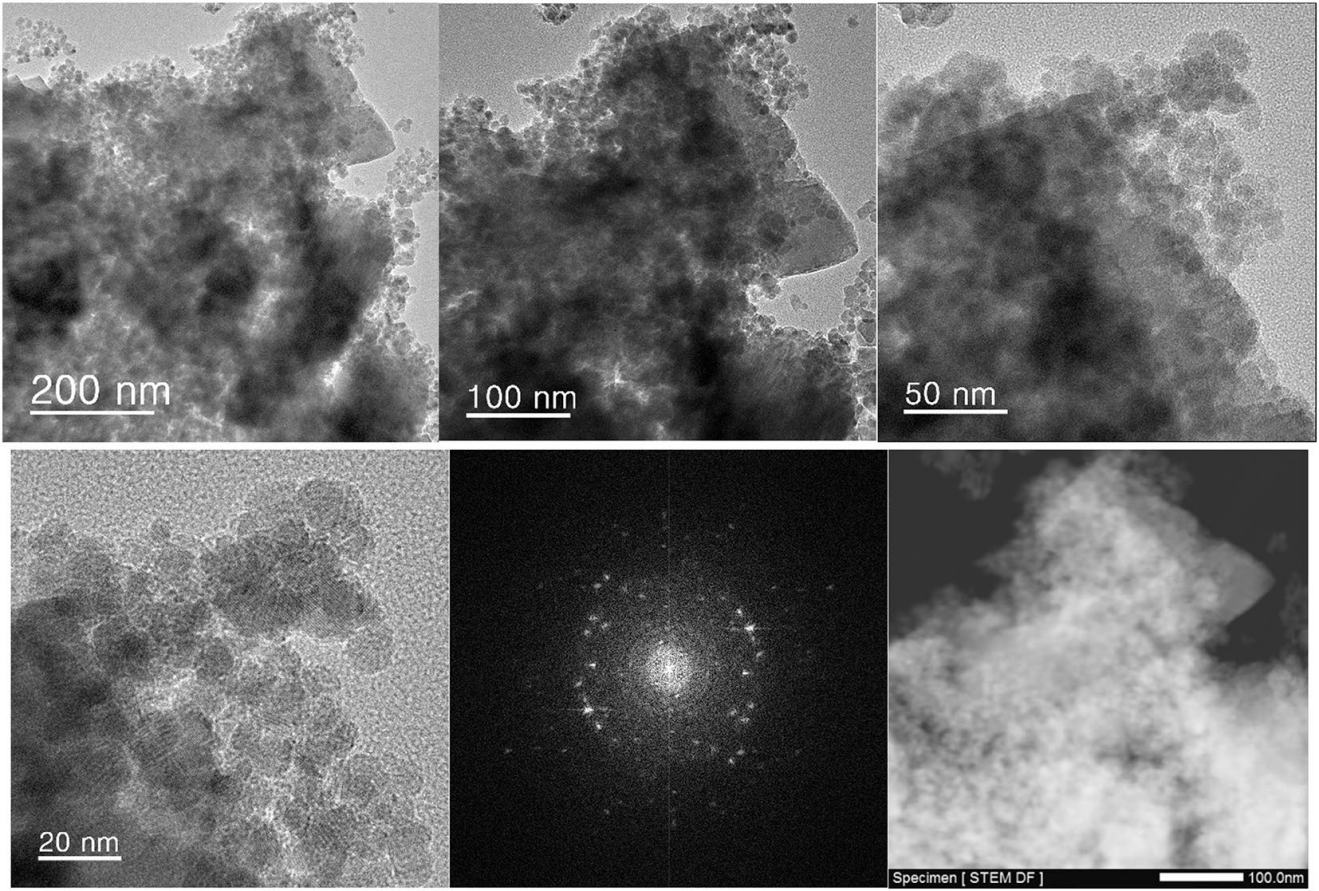
TEM, HRTEM, FFT and STEM images of Ps@Tet-Cu(II)@Fe_3_O_4_.

The TEM analysis of Ps@Tet, Ps@Tet@Fe_3_O_4_ and Ps@Tet-Cu(II)@Fe_3_O_4_ was applied to confirm the formation of Cu NPs on the surface of Ps@Tet@Fe_3_O_4_ (Figs. 3, 4, 5). As observed in Figs. 3, 4, 5, Cu NPs have been successfully loaded on the Ps@Tet@Fe_3_O_4_. The TEM and HRTEM images illustrate the fine dispersion of Cu NPs with the size of 8–10 nm on the Ps@Tet@Fe_3_O_4_ surface, accumulated in sites corresponding to iron oxide NPs. The HRTEM and FFT images of the Ps@Tet-Cu(II)@Fe_3_O_4_ show that the nanoparticles are highly crystalline. The STEM image confirms a homogeneously assembled nanostructured catalyst (Figs. 4 and 5).

**Figure 6:**
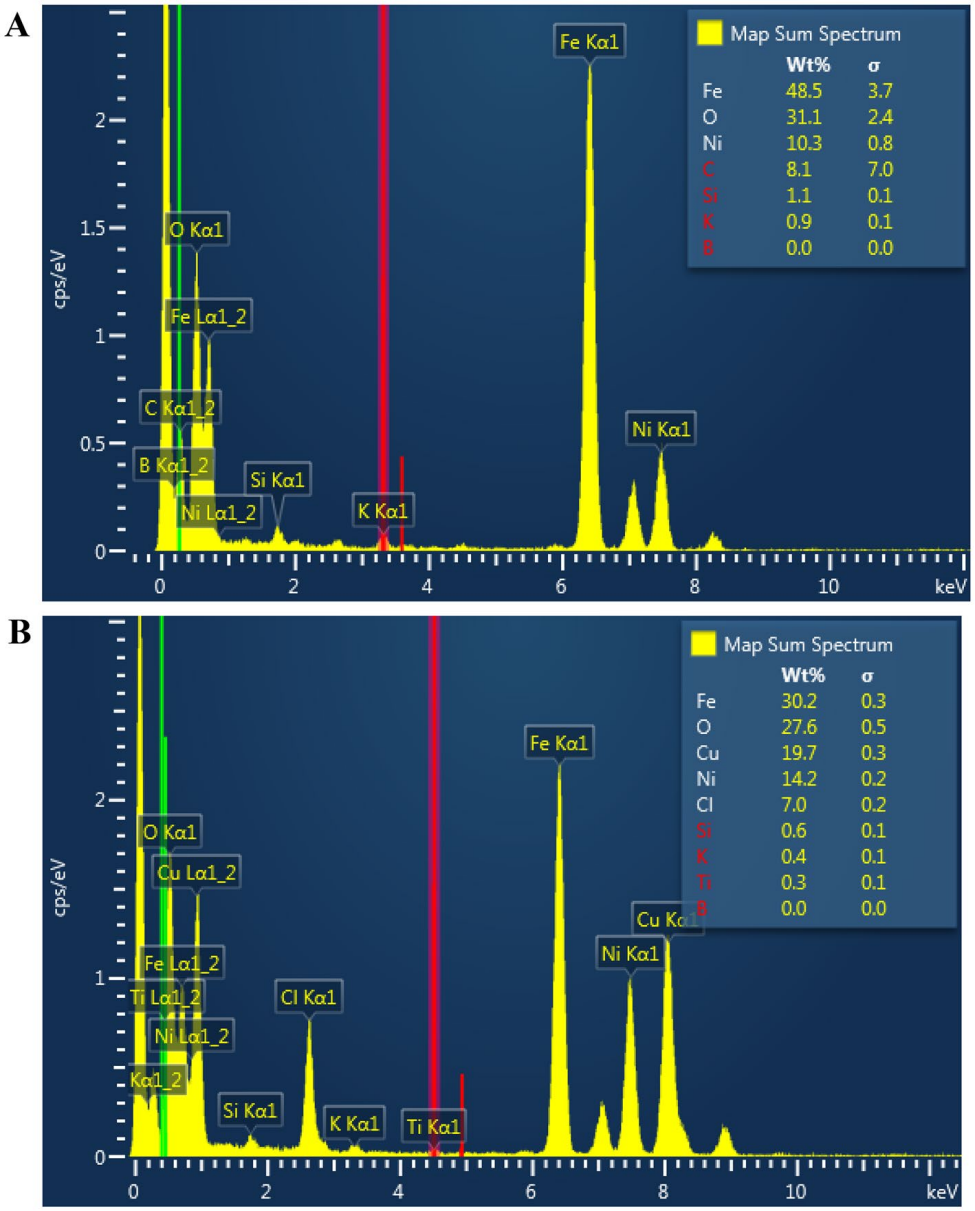
EDS spectra of Ps@Tet@Fe_3_O_4_ (**A**) and Ps@Tet-Cu(II)@Fe_3_O_4_ (**B**).

The EDS spectroscopy was used to determine the composition of Ps@Tet@Fe_3_O_4_ and Ps@Tet-Cu(II)@Fe_3_O_4_ complex (Fig. 6). The EDS analysis shows the presence of desired elements in their chemical structure. Figure 6 confirms that C, O, Si, and Fe are the main components present in both Ps@Tet@Fe_3_O_4_ and Ps@Tet-Cu(II)@Fe_3_O_4_ along with Cu and Cl elements, which are present only in the Ps@Tet-Cu(II)@Fe_3_O_4_ complex, further reaffirming the formation of the final catalyst. The amount of Cu incorporated into the Ps@Tet-Cu(II)@Fe_3_O_4_ complex was found to be 19.7 w%, as measured by EDS. According to ICP-OES analysis, the amount of Cu is 7.6 wt.%.

**Figure 7:**
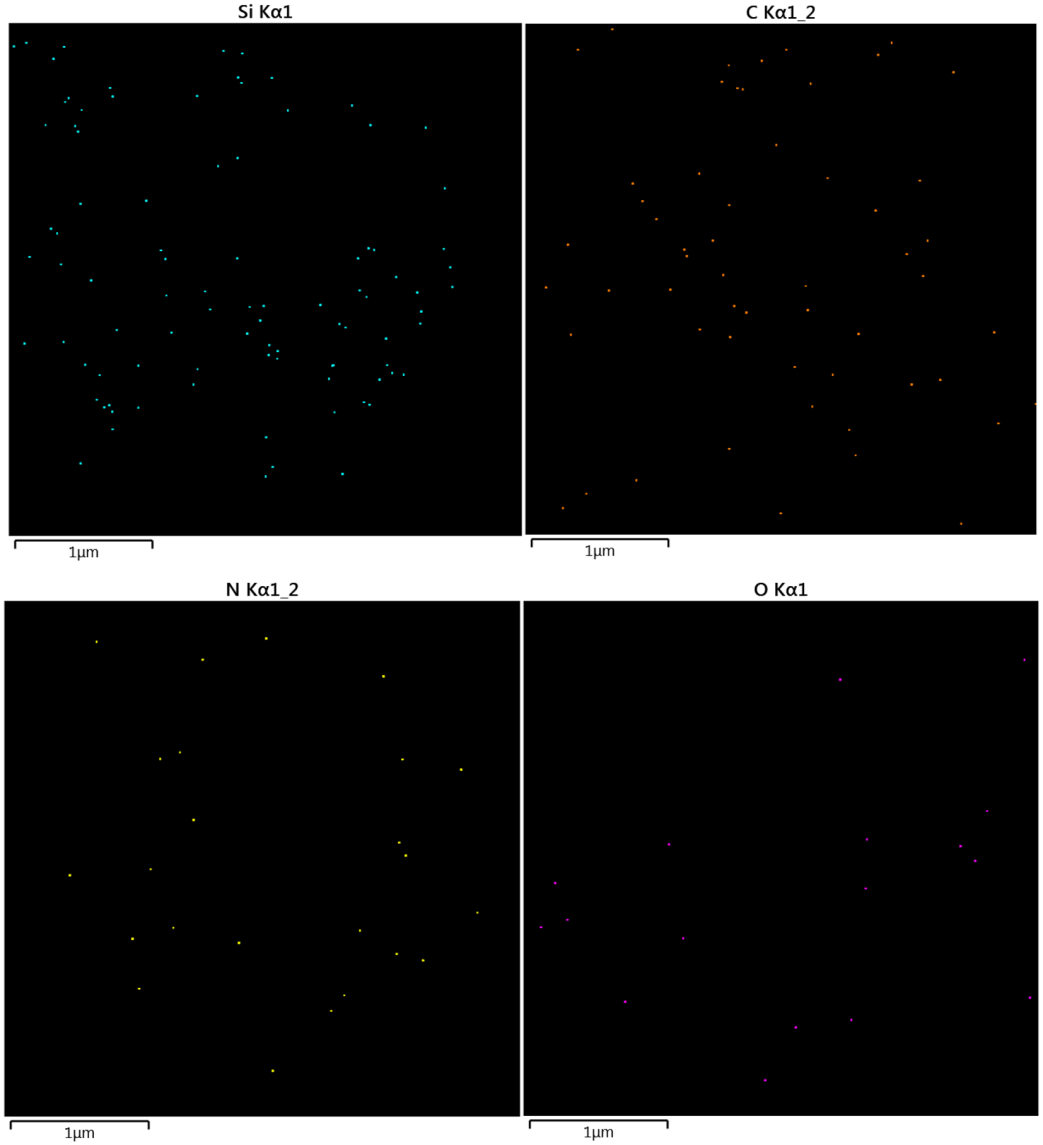
Elemental mapping of Ps@Tet.

**Figure 8:**
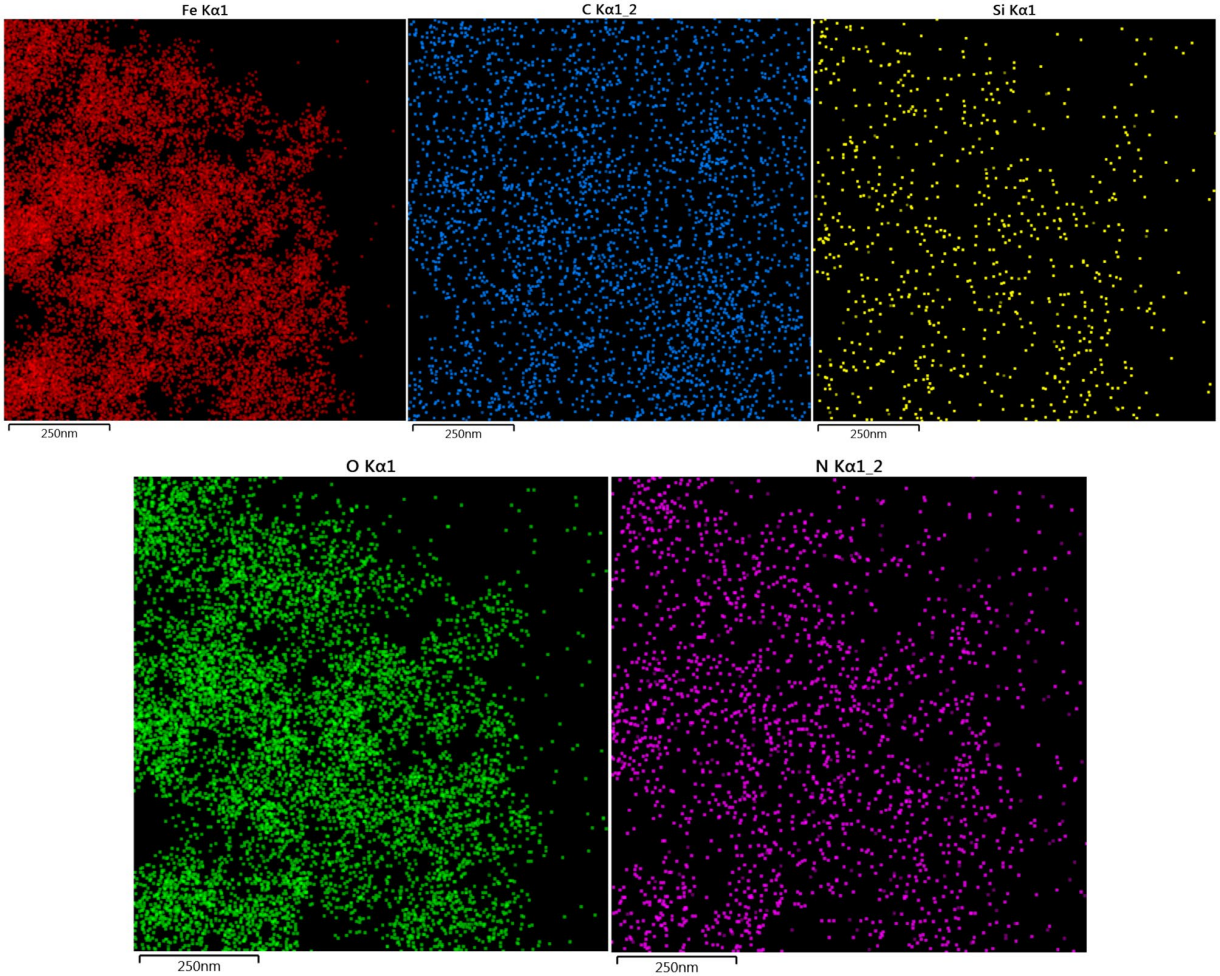
Elemental mapping of Ps@Tet@Fe_3_O_4_.

**Figure 9:**
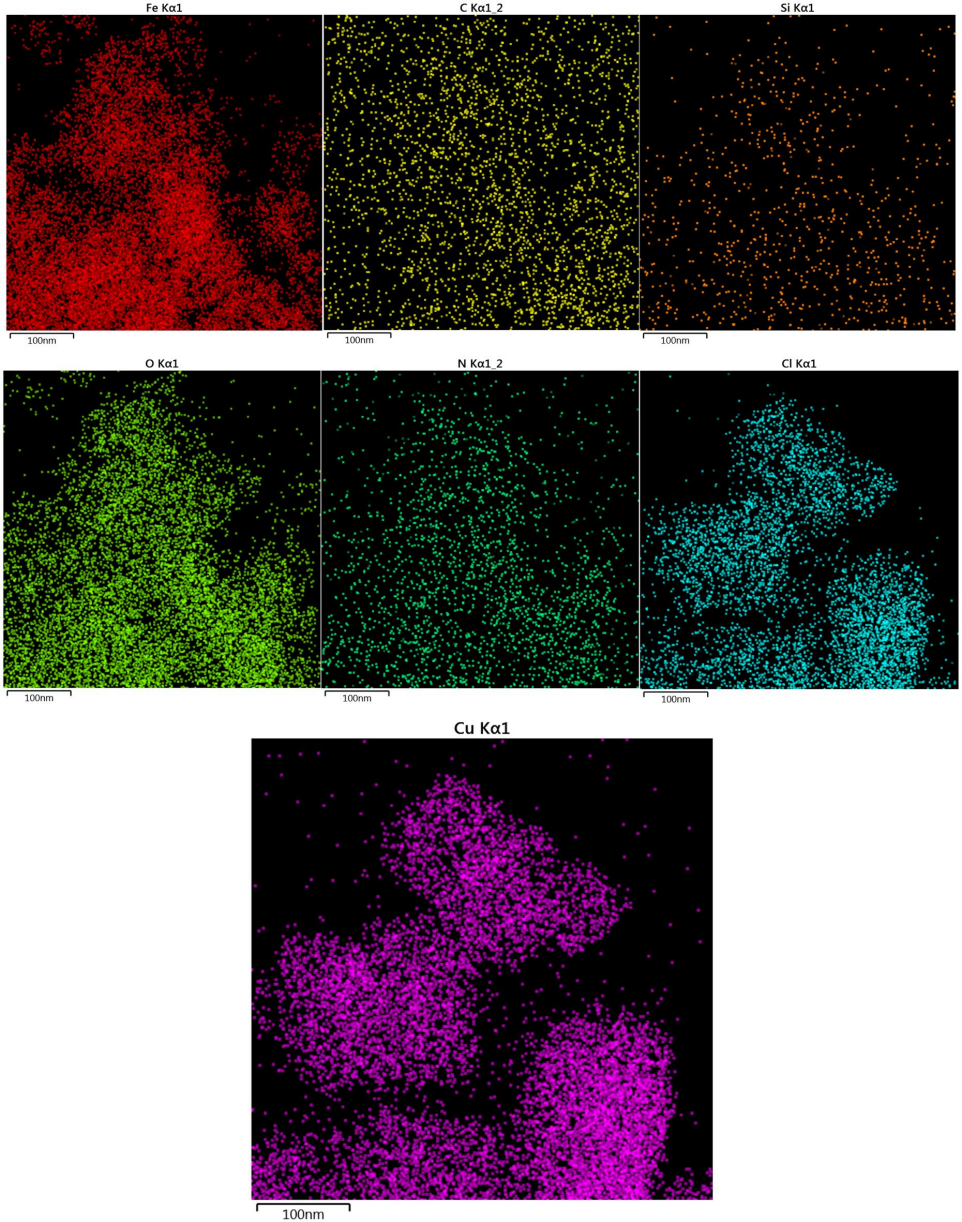
Elemental mapping of Ps@Tet-Cu(II)@Fe_3_O_4_.

Elemental mapping of Ps@Tet, Ps@Tet@Fe_3_O_4_, and Ps@Tet-Cu(II)@Fe_3_O_4_ are presented in Figs. 7, 8, 9. Elemental mapping was performed to determine the distribution of the elements on Ps@Tet-Cu(II)@Fe_3_O_4_ complex surface. Figures 7, 8, 9 confirm that C, O, Si, and N are main components present in Ps@Tet, Ps@Tet@Fe_3_O_4_, and Ps@Tet-Cu(II)@Fe_3_O_4_, along with Fe element, which was present only in the Ps@Tet@Fe_3_O_4_ and Ps@Tet-Cu(II)@Fe_3_O_4_ (Figs. 8 and 9). Additionally, the presence of Cl and Cu was determined using elemental mapping (Fig. 9); which indicated the uniform dispersion of Cu on the Ps@Tet@Fe_3_O_4_ surface.

**Figure 10:**
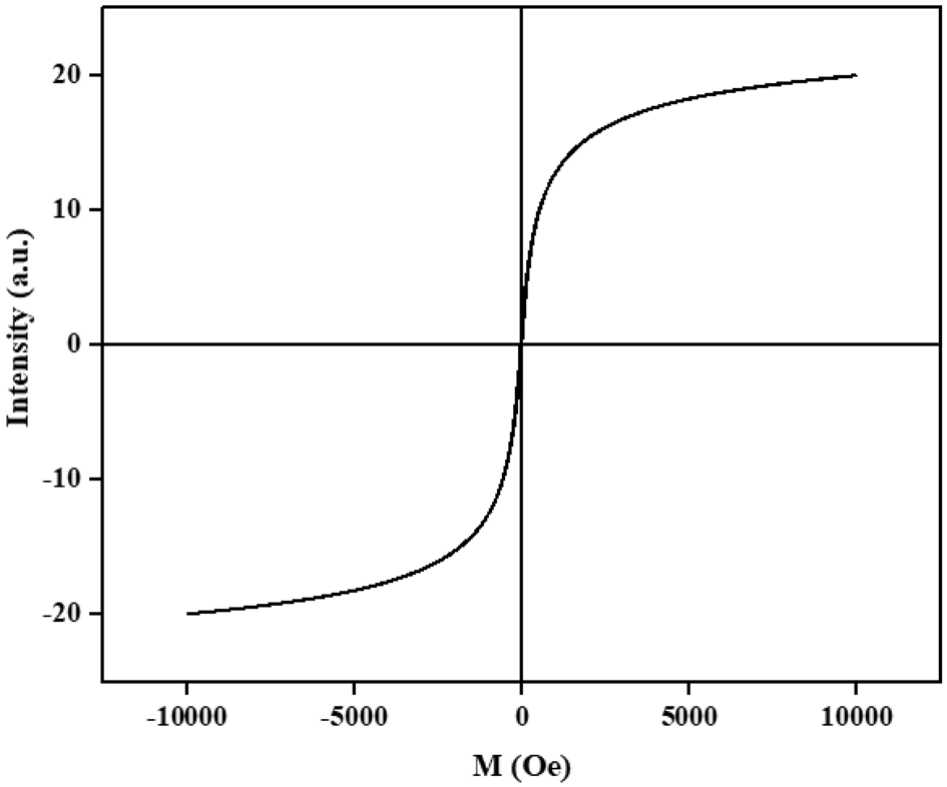
VSM analysis of Ps@Tet-Cu(II)@Fe_3_O_4_.

The magnetic properties of the synthesized Ps@Tet-Cu(II)@Fe_3_O_4_ complex were studied using VSM, as shown in Fig. 10. The specific saturation magnetization values (Ms) were calculated to be 60 and 20 emu/g for Fe_3_O_4_ NPs and Ps@Tet-Cu(II)@Fe_3_O_4_ complex, respectively, indicating that the modification of the surface and the addition of portions have led to decreased saturation magnetizations. Therefore, this complex has superparamagnetic characteristics and high magnetization values, enabling its separation by an external magnet from the reaction mixture.

**Figure 11:**
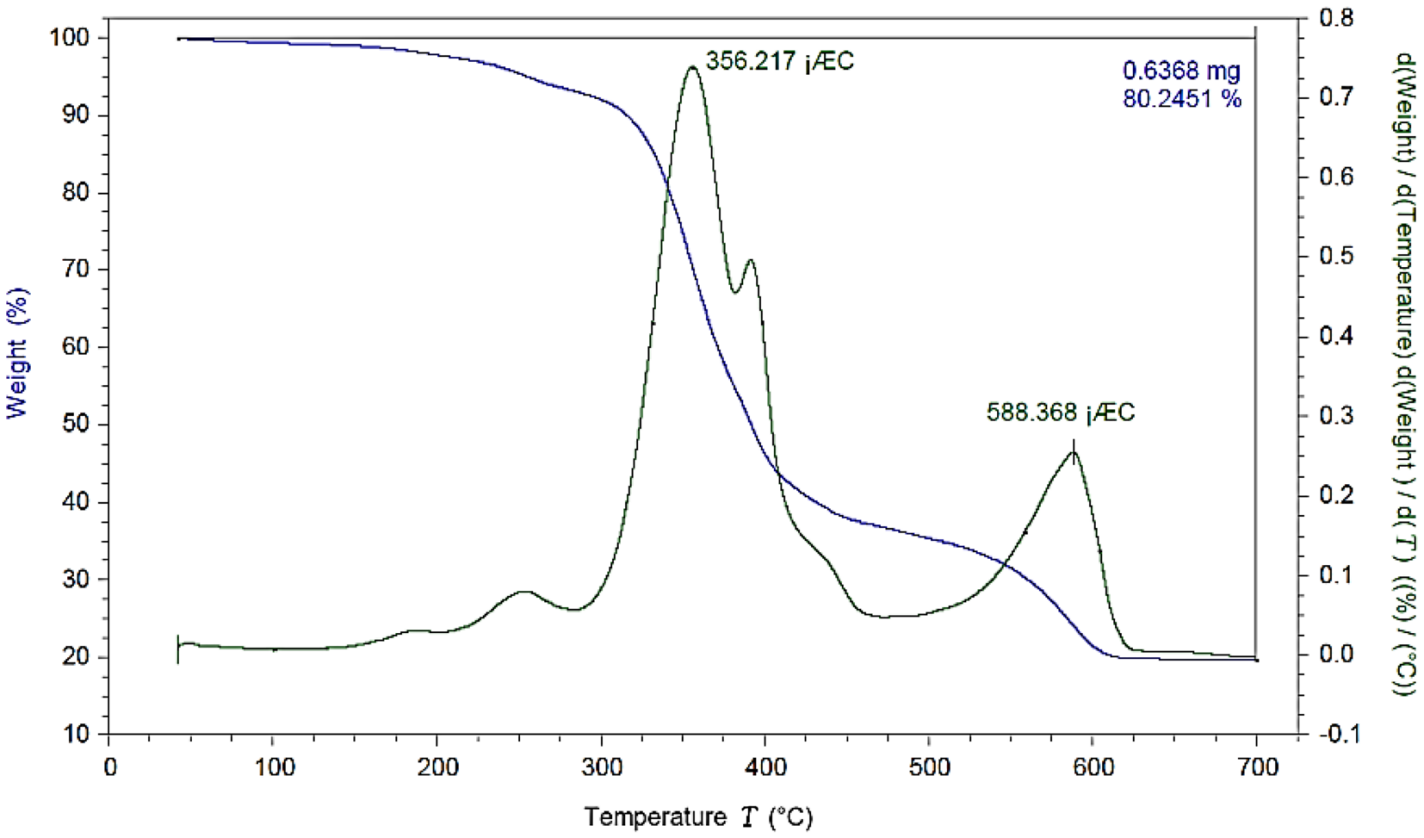
TG/DTG analysis of Ps@Tet-Cu(II)@Fe_3_O_4_.

The TG/DTG analysis is a great technique to measure thermal stability. Therefore, the thermal stability of the synthesized complex was checked over a temperature range of 30–700 ℃ (Fig. 11). The polymer-supported Cu(II) complex is stable up to 300 ℃. The first step of degradation (up to 300 ℃) is due to the removal of water and organic solvents. The second mass reduction is related to the degradation of organic groups such as 5-amino-1*H*-tetrazole in the temperature range of 300–410 ℃. The final degradation stage corresponds to the complete decomposition of functional groups of the catalyst. This degradation occurs when the temperature increases from 500 to 600 ℃.

### Synthesis of *N*-sulfonyl-*N*-aryl tetrazoles

The catalytic performance of Ps@Tet-Cu(II)@Fe_3_O_4_ was investigated in the [2 + 3] cycloaddition reaction. The synthesis of *N*-sulfonyl-*N*-aryl tetrazoles by the reaction of *N*-sulfonyl-*N*-aryl cyanamide and NaN_3_ as a model reaction in the presence of Ps@Tet-Cu(II)@Fe_3_O_4_ complex as a novel catalyst was studied for this purpose.

In the first step, the optimization of the reaction conditions was performed using *N*-(4-chlorophenyl)-*N*-cyano-4-methylbenzenesulfonamide (1 mmol) as a model substrate, NaN_3_ (1.5 mmol), Ps@Tet-Cu(II)@Fe_3_O_4_ complex and DMF solvent at 120 ℃. The results of the optimization reactions are shown in Table 1. As observed, the reaction does not proceed in the absence of the catalyst.

**Table 1:** Optimization of reaction conditions^a^.


Entry	Catalyst (g)	Time (min)	Yield^b^ (%)
1	0	100	0
2	0.01	65	69
3	0.03	45	78
4	0.05	25	86
5	0.07	25	86

^a^ Reaction conditions: *N*-(4-chlorophenyl)-*N*-cyano-4-methylbenzenesulfonamide (1 mmol), NaN_3_ (1.5 mmol), Ps@Tet-Cu(II)@Fe_3_O_4_, DMF (10 mL), 120 ℃.

^b^ Isolated yield.

After the optimization of the reaction, the efficiency of Ps@Tet-Cu(II)@Fe_3_O_4_ complex for the synthesis of various derivatives of *N*-sulfonyl-*N*-aryl tetrazole using various types of *N*-sulfonyl-*N*-aryl cyanamides containing electron-withdrawing as well as electron-donating groups was investigated (Table 2). Both groups on the aromatic ring of *N*-sulfonyl-*N*-aryl cyanamides favor the formation of the resulting target tetrazoles in high yields and short reaction times.

**Table 2:** Synthesis of tetrazoles using Ps@Tet-Cu(II)@Fe_3_O_4_ complex.^a^


Entry	Initial substance	Product	Time (min)	Yield^b^ (%)	TON	TOF (min^−1^)
1			25	86	14,405	576
2			30	85	14,237	474
3			30	82	13,735	458
4			30	84	14,070	469
5			35	82	13,735	392
6			30	83	13,902	463
7			30	86	14,405	480
8			30	84	14,070	469

^a^Reaction conditions: *N*-sulfonyl-*N*-aryl cyanamide (1 mmol), NaN_3_ (1.5 mmol), Ps@Tet-Cu(II)@Fe_3_O_4_ (0.05 g), DMF (10 mL), 120 ℃.

^b^ Isolated yield.

**Scheme 3:**
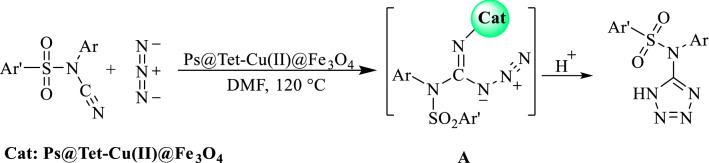
Proposed mechanism for the synthesis of tetrazoles.

The proposed mechanism for the synthesis of tetrazoles using Ps@Tet-Cu(II)@Fe_3_O_4_ complex is presented in Scheme 3. According to the reaction procedure, initially, an interaction occurs between the CN group of *N*-sulfonyl-*N*-aryl cyanamides in the presence of Ps@Tet-Cu(II)@Fe_3_O_4_ complex. Next, N_3_^-^ addition to the activated CN group gives the intermediate (**A**). Finally, the intramolecular cyclization of (**A**) leads to the desired product. This method has merits including high yields, short reaction time, and lack of production of HN_3_ toxic gas^85^.

### Summary and discussion

*N*-Sulfonyl-*N*-aryl tetrazole derivatives are very new compounds synthesized and reported by our research groups in recent years. In two previous publications, the synthesis of these novel derivatives through different reaction conditions have been reported. For example, for the first time, the synthesis of *N*-sulfonyl-*N*-aryl tetrazole derivatives was carried out in the presence of NaN_3_, ZnBr_2_, and H_2_O under reflux conditions for 24 h^85^. Although the product yields were relatively good, the reaction time was very long. In another study, the synthesis of these derivatives using Cu NPs@Fe_3_O_4_-chitosan catalyst, NaN_3_, and H_2_O under reflux conditions was investigated^66^. The drawback of the latter synthesis procedure was still the long reaction time (22 h). In addition, in our recent study, the synthesis of *N*-sulfonyl-*N*-aryl tetrazole derivatives using magnetic chitosan functionalized trichlorotriazine-5-amino-1*H*-tetrazole copper(II) complex catalyst and DMF solvent under reflux conditions has been reported^67^. The reaction suffered from long reaction time (40 min). Nevertheless, in the present work, *N*-sulfonyl-*N*-aryl tetrazole derivatives have been synthesized with high efficiency (82–86%) and in very short reaction times (25–35 min).

### Catalyst recyclability

**Figure 12:**
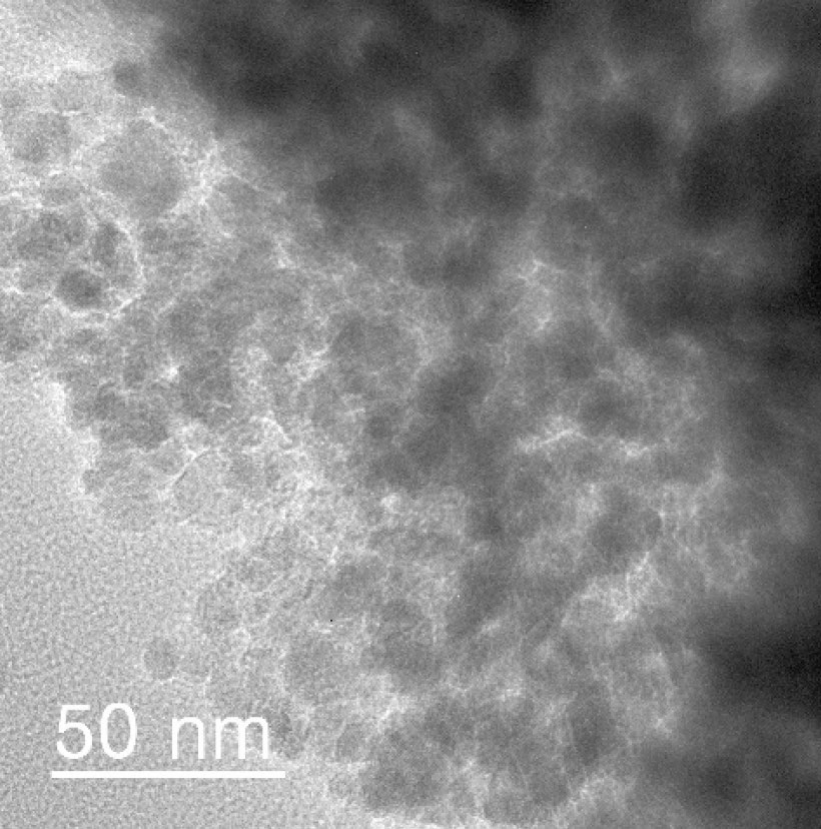
TEM image of the recycled Ps@Tet-Cu(II)@Fe_3_O_4_.

Reusability of heterogeneous catalysts is the most important advantage for practical purposes; especially for industrial applications. After completing the reaction, this magnetic complex was separated easily from the reaction media by an external magnet, washed with ethanol, dried, and reused for the same reaction without any significant reduction in the desired yields. Ps@Tet-Cu(II)@Fe_3_O_4_ exhibited a high activity over five runs, which confirms the catalyst stability. After the last run, the characterization of the recovered catalyst by TEM analysis (Fig. 12) showed a stable morphology and relatively dispersed NPs even after five runs as well as the stable structure of the recycled catalyst. To check the heterogeneity of Ps@Tet-Cu(II)@Fe_3_O_4_ catalyst, the filtrate of each cycle was analyzed by ICP-OES analysis. It was shown that less than 0.1% of the total amount of the original copper species was lost in the solution during a reaction.

## Conclusions

A novel, easily recoverable, and suitable heterogeneous catalyst has been developed for the synthesis of *N*-sulfonyl-*N*-aryl tetrazole derivatives. The significant advantages of Ps@Tet-Cu(II)@Fe_3_O_4_ complex as a magnetic nanocatalyst are its high surface area, simple separation, and outstanding stability. Afterward, the morphology and structure of the synthesized complex were investigated using TEM, HRTEM, STEM, FFT, XRD, FT-IR, TG/DTG, VSM, EDS, and elemental mapping. The catalytic activity of the obtained complex for the synthesis of *N*-sulfonyl-*N*-aryl tetrazole derivatives was checked. The advantages of the method include easy work-up, high yields, and avoidance of the use of harmful and hazardous hydrazoic acid. The magnetic nanocatalyst is environmentally friendly and commercial because it can be recovered using an external magnet and reused in the same reaction without considerable loss of catalytic activity.

## Data Availability

The datasets used and/or analyzed during the current study are available from the corresponding author on reasonable request.
